# Severe COVID-19 and breakthrough infections in vaccinated schizophrenia patients: A matched controlled cohort study

**DOI:** 10.1192/j.eurpsy.2023.326

**Published:** 2023-07-19

**Authors:** D. Tzur Bitan

**Affiliations:** Department of Psychology, Ariel University, Ariel; Psychiatric ER, Shlavata MHC, Hod Hasharon, Israel

## Abstract

**Introduction:**

Schizophrenia patients are at an increased risk for severe SARS-CoV-2 illness. Recent studies indicate that vaccines reduce morbidity gaps between schizophrenia patients and the general population; nonetheless, the ongoing emergence of COVID-19 variants and the increased frequency of breakthrough infections might lead to changes in these risk profiles.

**Objectives:**

In this study we aimed to bridge this gap by assessing the risk of COVID-19 infection, hospitalization, and mortality among vaccinated individuals with schizophrenia, as compared to vaccinated individuals with no schizophrenia, matched for age, sex, and vaccination coverage (first, second, and booster) throughout the first year of the vaccination plan.

**Methods:**

The study included 50,958 vaccinated individuals: 25,479 individuals with schizophrenia and 25,479 without schizophrenia. Data were derived from the databases of Clalit Health Services, the largest healthcare organization in Israel.

**Results:**

Findings indicated that differences among vaccinated schizophrenia patients and controls were non-significant after adjusting for infection (HR = 0.93, 95%CI 0.84-1.03, p = 0.14) and mortality rates (HR = 2.18, 95%CI 0.80-5.90, p = 0.12). Nonetheless, differences in rates of hospitalization remained significant even after controlling for demographic and clinical factors (HR = 2.68, 95%CI 1.75-4.08, p<0.001). A longitudinal assessment of relative risk indicated that the rate ratio of differences between the groups increased during the fourth infection wave of the B.1.617.2 (delta) variant across all parameters, with schizophrenia patients demonstrating higher relative risk of hospitalization (RR = 4.19, 95%CI 2.41-7.23) and mortality (RR = 7.61, 95%CI 0.93-61.89) during the relevant periods.

**Conclusions:**

These findings suggest that vaccination coverage is effective in narrowing overall morbidity and mortality gaps; nonetheless, individuals with schizophrenia are still at risk for severe COVID-19 outcomes.

**Disclosure of Interest:**

None Declared

